# Technology-Based Interventions in Oral Anticoagulation Management: Meta-Analysis of Randomized Controlled Trials

**DOI:** 10.2196/18386

**Published:** 2020-07-15

**Authors:** Hengfen Dai, Caiyun Zheng, Chun Lin, Yan Zhang, Hong Zhang, Fan Chen, Yunchun Liu, Jingwen Xiao, Chaoxin Chen

**Affiliations:** 1 Affiliated Fuzhou First Hospital of Fujian Medical University Fuzhou China; 2 Fuqing City Hospital Fuzhou China; 3 School of Basic Medical Sciences Fujian Medical University Fuzhou China; 4 School of Pharmacy Fujian Medical University Fuzhou China

**Keywords:** technology-based, oral anticoagulation management, meta-analysis, randomized controlled trials, telehealth, warfarin

## Abstract

**Background:**

An increasing number of patients have received prophylactic or therapeutic oral anticoagulants (OACs) for thromboembolic complications of diseases. The use of OACs is associated with both clinical benefits and risks. Considering the challenges imposed by this class of drugs, as well as the enormous progress made in portable device technology, it is possible that technology-based interventions may improve clinical benefits for patients and optimize anticoagulation management.

**Objective:**

This study was designed to comprehensively evaluate the role of technology-based interventions in the management of OACs.

**Methods:**

We searched 6 databases—PubMed, EMBASE, Cochrane, Cumulative Index to Nursing and Allied Health Literature, Scopus, and PsycINFO—to retrieve relevant studies published as of November 1, 2019, to evaluate the effect of technology-based interventions on oral anticoagulation management. RevMan (version 5.3; Cochrane) software was used to evaluate and analyze clinical outcomes. The methodological quality of studies was assessed by the Cochrane risk of bias tool.

**Results:**

A total of 15 randomized controlled trials (RCTs) were selected for analysis. They reported data for 2218 patients (1110 patients in the intervention groups and 1108 patients in the control groups). A meta-analysis was performed on the effectiveness and safety data reported in the RCTs. Technology-based interventions significantly improved the effectiveness of oral anticoagulation management (mean difference [MD]=6.07; 95% CI 0.84-11.30; I^2^=72%; *P*=.02). The safety of oral anticoagulation management was also improved, but the results were not statistically significant. Bleeding events were reduced (major bleeding events MD=1.02; 95% CI 0.78-1.32; I^2^=0%; *P*=.90; minor bleeding events MD=1.06, 95% CI 0.77-1.44; I^2^=41%; *P*=.73) and thromboembolism events were reduced (MD=0.71; 95% CI 0.49-1.01; I^2^=0%; *P*=.06). In general, patients were more satisfied with technology-based interventions, which could also improve their knowledge of anticoagulation management, improve their quality of life, and reduce mortality and hospitalization events.

**Conclusions:**

Using technology to manage OACs can improve the effectiveness and safety of oral anticoagulation management, result in higher patient satisfaction, and allow greater understanding of anticoagulation.

## Introduction

### Oral Anticoagulation Management

Oral anticoagulants (OACs) have been used for decades, especially warfarin, which has been in use since the 1950s. Until recently warfarin has been a fundamental drug in clinical anticoagulant therapy, and although the results of two previously published meta-analyses of atrial fibrillation suggested that novel OACs are not inferior to warfarin in terms of safety and efficacy [1,2], warfarin has the advantage of being inexpensive and having a wide range of indications. Warfarin is widely used in conditions that are prone to thrombosis, such as atrial fibrillation, irregular heartbeat, myocardial infraction, artificial heart valve replacement, recurrent stroke, deep vein thrombosis, and pulmonary embolism [3]. However, it remains challenging to balance effectiveness and safety in treatment [4]. Statistical data from relevant literature estimated that over 6 million patients in the United States have received anticoagulant therapy [5], leading to increased risks of bleeding, thromboembolism, hospitalization, and mortality. Currently, rough statistics indicate that about 1 in 10 surgical patients in the United States receive OACs [6], and approximately 2 million people start warfarin therapy in the United States every year [7]. Therefore, effective anticoagulation management measures are urgently needed.

### Technology-Based Interventions for Oral Anticoagulation Management

With the continuous development of telemedicine health service technology, more and more technological devices have been applied to help in the management of patients with chronic diseases, including those with diabetes, hypertension, heart failure, and others [8]. Telemedicine is defined as a long-distance medical practice that can be characterized by health services through a wide range of technical applications and services [9]. It is a new way of providing high-quality resources to primary medical institutions through remote consultation on the basis of internet convenience. Through this approach, medical services and medical activities are mainly carried out through computers, various remote communications, medical technology, and medical equipment. These remote forms of communication enable contact between patients and medical personnel, medical institutions, and medical equipment. This process, in turn, can assist in diagnoses, treatment, monitoring, and follow-up [10]. As a result, telemedicine has become an increasingly popular model for providing accurate international normalized ratio (INR) monitoring for patients taking warfarin. Other terms used to describe telemedicine include connected health [11], mobile health (mHealth) [12], and electronic health (eHealth) [13], which are collectively called technology-based interventions [14]. There is interest in learning whether these interventions can assist medication management and enhance patient compliance, with results suggesting some benefit [15]. 

### Aim of the Study

Telemedicine technology has the potential to enhance multiple aspects of anticoagulant therapy management [16], including patient education, symptom monitoring, follow-up, and encouragement and tracking of medication adherence, given its accessibility by telephone, internet, voicemail messaging, and apps. However, no systematic review or meta-analysis has been published to summarize what is currently known on this topic. Thus, we aimed to evaluate the effectiveness and safety of technology-based interventions in OAC management by performing a meta-analysis.

## Methods

### Literature Search

Eligible studies were identified by searching PubMed, EMBASE, Cochrane, Cumulative Index to Nursing and Allied Health Literature, PsycINFO, Scopus, and other relevant databases, and the results were combined using literature traceability methods. Searches were conducted on November 1, 2019. Search keywords included *warfarin therapy+*, *oral anticoagulation management+*, *telephone*, *eHealth*, *apps*, and *telemedicine+*. Search strategies are detailed in Multimedia Appendix 1. Only papers written in English were considered. No restriction regarding publication date was applied.

### Inclusion and Exclusion Criteria

#### Inclusion Criteria

The studies included in the analysis met the following criteria: (1) the study was a randomized controlled trial (RCT); (2) the subjects were taking warfarin; (3) technology-based interventions were used to manage OACs; (4) results included the time in therapeutic range (TTR), bleeding, and thromboembolism events; and (5) the results were reported in English.

#### Exclusion Criteria

Exclusion criteria included: (1) studies that were retrospective, observational, reviews, model research, literature reviews, or conference summaries; (2) the results of the study did not involve the TTR, bleeding, and thromboembolism events; (3) the study was a duplicate report; and (4) technical intervention was only used as a means to collect data.

### Document Screening and Data Extraction

All references were initially screened by title and abstract by 2 reviewers for relevance. Finally, full-text analysis for eligibility was performed independently by 2 authors, HD and CL. Disagreements were discussed and resolved by consensus or third-party arbitration.

The required data were extracted by a researcher using a literature data extraction table, and another researcher confirmed the accuracy and authenticity of the data. The extracted content included (1) basic information of study, such as research topic, author, and date; (2) baseline characteristics of the study subjects, such as sample size, median age, and sex; (3) follow-up time for interventions; (4) efficacy and safety information after interventions, such as TTR, bleeding, and thromboembolism events; and (5) other outcome indicators, including time within expanded target INR range, mortality, and hospitalization events.

### Literature Quality Evaluation

Risk of bias assessment of the included RCTs was performed using the Cochrane risk of bias tool based on the *Cochrane Handbook for Systematic Reviews of Interventions*' literature evaluation criteria [17].

### Statistical Analysis

The meta-analysis of RCTs was performed using RevMan (version 5.3; Cochrane) software. Heterogeneity was assessed using a chi-square test, and quantitative analysis was performed using I^2^. Values of *P*≥.05 and I^2^≤50% were considered to represent no heterogeneity, in which case a fixed-effect model was used. If *P*<.05 and I^2^>50%, a random-effects model was used [18].

## Results

### Search Results

**Figure 1:**
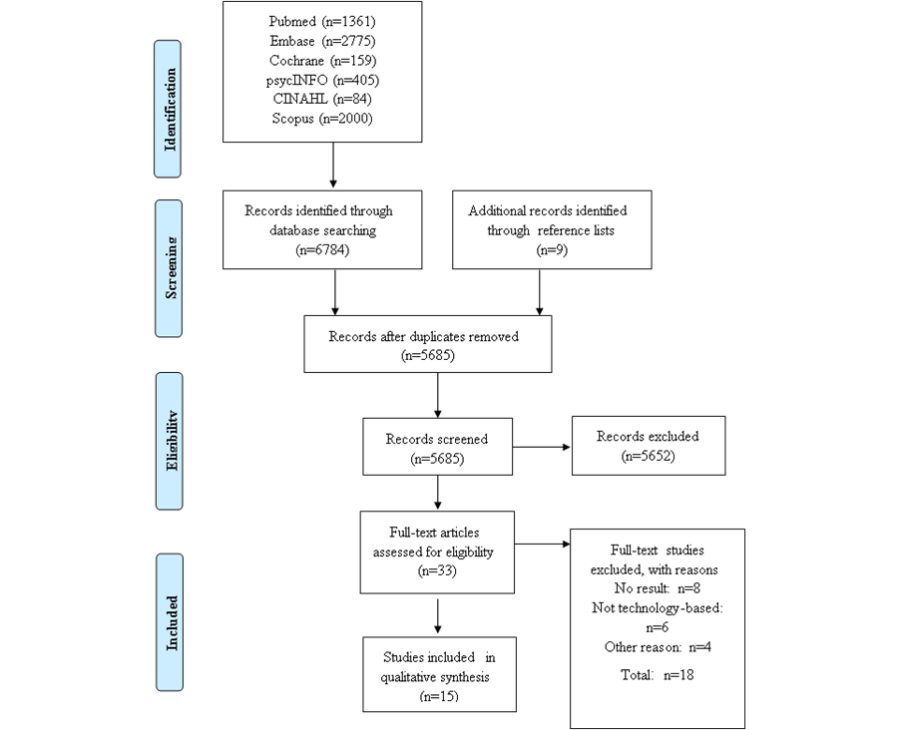
Flow chart of the systematic literature review process. CINAHL: Cumulative Index to Nursing and Allied Health Literature.

A total of 6784 papers were retrieved from the systematic literature search, and 9 papers were retrieved by other means, totaling 6793 papers. After removing duplicate studies, 2 authors independently reviewed and excluded another 5652 studies that did not meet predetermined selection criteria, based on the title and abstract of each paper. After reading 33 eligible full-text studies, 18 were excluded and 15 were selected for inclusion in the analysis [12,19-31]. The systematic search results are shown in Figure 1.

### Basic Characteristics and Quality Evaluation of the Literature

**Figure 2:**
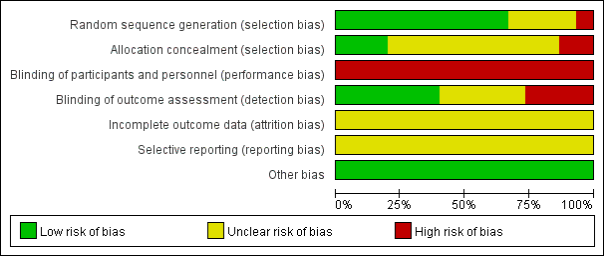
Bias risk map from the Cochrane systematic evaluation method to evaluate the quality of the included randomized controlled trials.

The 15 RCTs included a total of 2218 patients: 1110 patients in the technology-based intervention group and 1108 patients in the traditional intervention group. General information (such as sample size, median age, sex, and specific intervention measures) from the studies is shown in Table 1. The Cochrane systematic evaluation method was used for quality evaluation, and overall, the included studies had low risk of bias and were high quality, as shown in Figure 2.

**Table 1:** Baseline characteristics of the included studies.

Study	Primary indication for therapy (n)	N^a^	Age in years, mean (SD)	Men, n (%)	Follow-up duration, months	Intervention	Description of technology-based interventions
Beyth 2000 [19]	VTE^b^ (124) AF^c^ (54) Cerebrovascular disease (49) HVR^d^ (36) Others (41) PVD^e^ (14) Myocardial infarction (7)	325	74.7 (6.75)	141 (43.38)	6	telephone	Recommendations for dose and subsequent INR^f^ testing
Sidhu 2001 [20]	HVR (83)	83	60.9 (—)	46 (55.42)	24	telephone	Medical advice if patient’s INR was too high (>4.0) or too low (< 1.5) Medical advice for any bleeding or thromboembolic events
Fitzmaurice 2002 [21]	—	49	66 (—)	37 (75.51)	6	software	Medical advice to override the dosing algorithm
Khan 2004 [22]	AF (79)	79	74 (—）	45 (56.96)	6	telephone	Recommendations for dose
Staresinic 2006 [23]	AF (79) VTE (23) Cerebrovascular accident (19) Coronary artery disease (12) PVR^g^ (36) Others (23)	192	69.3 (9.1)	187 (97.40)	36	interim telephone	Telephone follow-up
Chan 2006 [24]	AF (72) HVR (24) DVT (17) PE^h^ (9) Cerebrovascular accident (4) Valvular heart diseases (5) Cardiomyopathy (2) Miscellaneous (4)	137	59 (14)	62 (45.26)	24	telephone	Consultation for difficult INR control or adherence issues
Lalonde 2008 [25]	AF (149) DVT^i^ (68) PE (26) Stroke (11) Cardiomyopathy (9) Myocardial infarction (8) Others (12)	250	65.45 (11.75)	128 (51.20)	6	telephone	Contact with pharmacist
Soliman 2008 [26]	Elective mechanical aortic valve replacement (—)	58	56 (8.95)	—	12	internet	Verify the anticoagulant dose on the website Pass the anticoagulant dosage exam on the website
Schillig 2011 [27]	VTE (100) AF (302) Others (98)	500	66.05 (15.25)	276 (55.20)	1	telephone	Contact responsible physician and anticoagulation clinic that provided dosing regimen
Verret 2012 [28]	AF or flutter (58) PVR (—)	114	57.7 (10.5)	78 (68.42)	4	voicemail message	Communicate Provide INR results and perform necessary adjustments
Bungard 2012 [29]	AF (49) VTE (8) Others (5)	62	73 (—)	38 (61.29)	6	telephone	Discuss any potential factors that may influence the INR result Warfarin dosing instructions Schedule a follow-up phone call
Lakshmi 2013 [30]	Mitral valve replacement (16) AF (45) DVT (2) PE (2) Valvotomy (1) Bioprosthetic valve (2) Other cardiac risk (12)	80	55.97 (12.85)	52 (60.00)	6	telephonic contact	Call the clinical pharmacist for clarification on any anticoagulation-related issues
Brasen 2018 [13]	AF (56) DVT/PE (14) Valvular heart disease (2) Various diagnoses (cardiomyopathy, aneurism, thrombophilia, and stroke) (15)	87	69.4 (—)	69 (79.30)	10	telemedicine software	Physician could inform patient of result, new dosage, and date for next INR measurement
Ayutthaya 2018 [31]	Valvular heart disease (14) Mechanical prosthetic valves (3) AF (31) DVT (14) PE (1)	50	57.65 (10.95)	30 (60.00)	3	telephone	Pharmacists perform medicine use review by asking patients about problems/obstacles with managing warfarin, including adverse events and complications Assess medication adherence Provide reminders for the next scheduled visits
Liang 2019 [32]	Non-valvular AF (80) Valvular AF (8) DVT (30) PE (12) Others (13) Multiple indications (9)	152	61.3 (15.4)	85 (55.92)	6	telephone	Pharmacists mainly assessed and reinforced adherence to warfarin and INR monitoring Education and recommendations according to participants’ recent INR assessment, self-reported medication or dietary changes, and anticoagulation-related complications

^a^N: total number of participants in the study.

^b^VTE: venous thromboembolism.

^c^AF: atrial fibrillation.

^d^HVR: heart valve replacement.

^e^PVD: peripheral vascular disease.

^f^INR: international normalized ratio.

^g^PVR: prosthetic valve replacement.

^h^PE: pulmonary embolism.

^i^DVT: deep vein thrombosis.

### Meta-Analysis

#### Time Within Target INR Range

**Figure 3:**
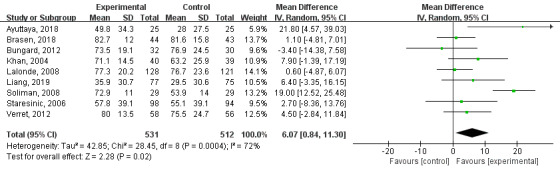
Results of a meta-analysis of the effects of technology-based interventions on time within target international normalized ratio range in oral anticoagulation management. IV: independent variable. Random: random effect model.

Of the 15 included studies, 9 included TTR values, for a total of 1043 patients. A random-effects analysis was used for the meta-analysis of these 9 RCTs. As shown in Figure 3, the TTR of the technology-based intervention group was significantly higher than that of the control group (mean difference [MD]=6.07; 95% CI 0.84-11.30; I^2^=72%; *P*=.02).

#### Bleeding Events

**Figure 4:**
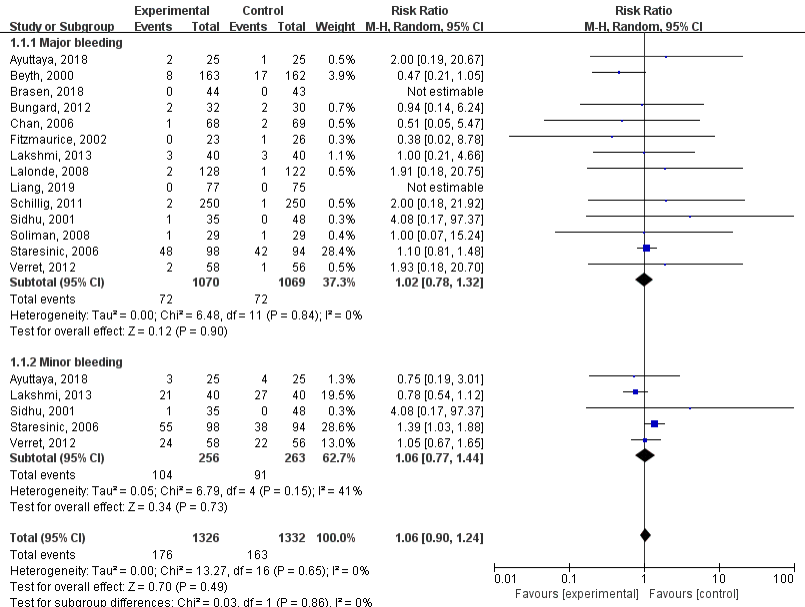
Results of a meta-analysis of the effects of technology-based interventions on major bleeding and minor bleeding events in oral anticoagulation management. M-H: Mantel-Haenszel method. Random: random effect model.

Of the 15 included studies, 14 included major bleeding values, for a total of 2139 patients. A random-effects model was used for the meta-analysis of these 14 RCTs. There were fewer major bleeding events in the technology-based intervention group than in the control group, but the difference was not statistically significant. (MD=1.02; 95% CI 0.78-1.32; I^2^=0%; *P*=.90). There were 5 papers that included minor bleeding values, for a total of 519 patients. A random-effects analysis model was used for the meta-analysis of these 5 RCTs. There were fewer minor bleeding events in the technology-based intervention group than in the control group, but the difference was not statistically significant (MD=1.06; 95% CI 0.77-1.44; I^2^=41%; *P*=.73). Major and minor bleeding event analyses are shown in Figure 4.

#### Thromboembolism Events

**Figure 5:**
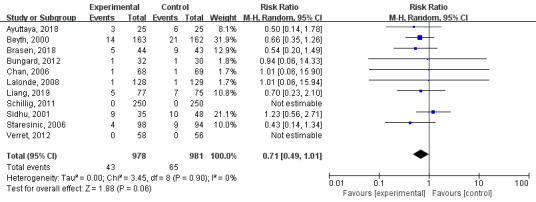
Results of a meta-analysis of the effects of technology-based interventions on thromboembolism events in oral anticoagulation management. M-H: Mantel-Haenszel method. Random: random effect model.

Of the 15 included papers, 11 had thromboembolism values, for a total of 1959 patients. A random-effects model was used for the meta-analysis of these 11 RCTs. As shown in Figure 5, there were fewer thromboembolism events in the technology-based intervention group than in the control group, but the difference was not statistically significant (MD=0.71; 95% CI 0.49-1.01; I^2^=0%; *P*=.06).

#### Sensitivity Analysis Results

The data were analyzed with fixed- and random-effects models, and the consistency of these results reflects the reliability of the combined results to some extent. The two effect models were used to analyze the combined effect of each risk factor and calculate 95% CIs. The results were similar, indicating that the results of this study are stable.

#### Other Results

**Figure 6:**
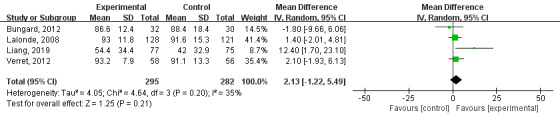
Results of a meta-analysis of the effects of technology-based interventions on time within extended target international normalized ratio range events in oral anticoagulation management. IV: independent variable. Random: random effect model.

**Figure 7:**
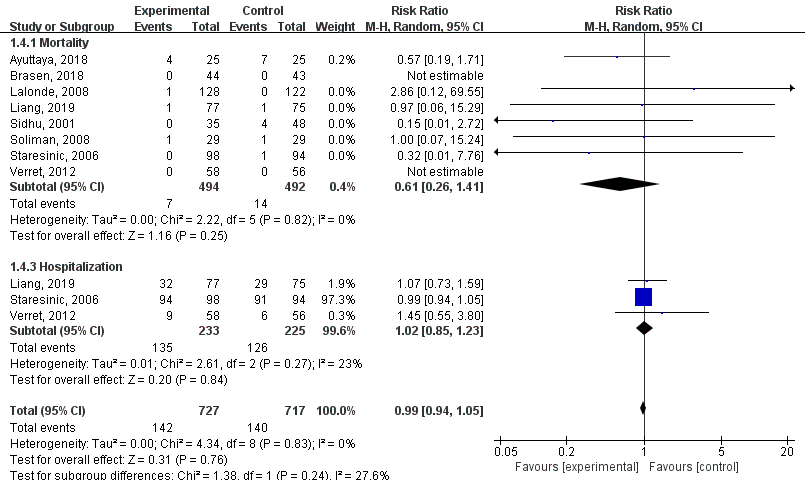
Results of a meta-analysis of the effects of technology-based interventions on mortality and hospitalization events in oral anticoagulation management. M-H: Mantel-Haenszel method. Random: random effect model.

The time within expanded target INR range of the technology-based intervention group was higher than that of the control group, but the difference was not statistically significant (MD=2.13; 95% CI –1.22 to 5.49; I^2^=35%; *P*=.21; Figure 6). There were fewer mortalities and hospitalization events in the technology-based intervention group than in the control group, but the difference was not statistically significant (mortality MD=0.61; 95% CI 0.26 to 1.41; I^2^=0%; *P*=.25; hospitalization MD=1.02; 95% CI 0.85 to 1.23; I^2^=23%; *P*=.84; Figure 7). There were 3 papers [28,30,32] that mentioned that patients' knowledge of anticoagulation was significantly improved through telemedicine intervention. There were 4 studies [22,25,26,28] that involved quality-of-life assessments; quality of life was higher in the intervention group than in the control group and it improved from baseline. There were 2 papers [21,25] that mentioned the higher costs in the technical intervention group than in the control group, and 1 paper [24] found that the costs for the nontechnical intervention group were higher. Patient satisfaction surveys were conducted in 5 studies [24,25,28-30], all of which indicated that the technical intervention group had higher satisfaction. There were 4 papers [25,26,28,32] that included data on INR tests, and the technical intervention group tended to have larger numbers of INR tests performed. 

## Discussion

### Principal Findings

We performed a meta-analysis of 15 RCTs to determine whether technology-based interventions were beneficial for patients compared with traditional interventions in oral anticoagulation management. TTR reflects the quality and effectiveness of warfarin and is an important determinant of bleeding and thromboembolism in anticoagulation management. A total of 9 studies were involved in the TTR analysis, and almost all studies reported that technology-based interventions were effective. The number of bleeding (including major and minor) events in the technology-based intervention groups was lower than that in the control groups, but the difference was not statistically significant. Outcomes were similar for the thromboembolism events. Time within expanded target INR range, mortality, and hospitalization were not significantly different between the technology-based management model and traditional management model. However, patients’ anticoagulation knowledge, patient satisfaction, quality of life, and number of INR tests performed were positively affected by technology-based management.

Oral anticoagulation management can be challenging when ensuring safety of the patient; as long as warfarin is used, regular monitoring is necessary [33]. The technology-based interventions used to manage oral anticoagulation typically include multiple methods, including telephone, internet, voicemail messaging, and apps. These tools are readily available in this era of technological advancement, and they reduce requirements for capacity and waiting time in outpatient clinics. Through this process, mHealth technologies have systematically demonstrated that they can help improve early diagnosis and treatment of atrial fibrillation and significantly reduce the cost of illness associated with atrial fibrillation [34]. One such example of mHealth technology is SintromacWeb, a management system of internet-based telecontrol tools that has been recognized to have better effectiveness and safety at oral anticoagulation therapy management than a conventional approach does [35]. Technology-based interventions can effectively and professionally teach patients about anticoagulation, provide interpretation of indicators, and provide a timely dose adjustment program.

### Comment on Results

It is worth mentioning that the mean age of the patients included in each study was over 60 years, so these patients may have had a relatively poor grasp on learning how to use novel websites and apps. The telephone is an ultra-portable electronic device, and it was chosen as the intervention tool in most of the 15 RCT studies included. Telephone intervention is especially suitable for older patients who are not accustomed to using computers or online services, or who may have difficulties in vision, finger dexterity, and mental state.

Our overall results showed that technology-based interventions significantly improved the effectiveness of oral anticoagulation management but did not significantly increase the safety or other results of oral anticoagulation management. These results may be because of the different follow-up times, which ranged from 1 month to 36 months. The greatest difference between the intervention and control groups in the frequency of major bleeding events occurred in the first week (when anticoagulation therapy was started in the hospital) and after the first month of therapy (when outpatient therapy was presumably stabilized). The 2 groups differed little during the second through fourth weeks of therapy, when patients were generally discharged and warfarin was first monitored in the outpatient setting [19]. That is to say, most patients might attach great importance to any type of intervention early on and then slowly relax later.

### Limitations

We acknowledge that our research has some limitations. The included studies had relatively small sample sizes and varying follow-up times, with some as short as 1 month. Warfarin is the oldest and best known OAC, and as long as warfarin is used, monitoring is necessary. Therefore, in order to more comprehensively assess the safety of OACs, longer follow-up times are needed. Furthermore, patients’ adherence to technology-based interventions may be lacking. It would be ideal if intervention compliance studies were added during follow-up. Despite these limitations, this study is the first to comprehensively analyze the effect of technology-based interventions on anticoagulation management. Therefore, larger sample sizes and longer clinical RCTs are needed to further evaluate the impact of technology-based interventions on anticoagulation management.

### Comparison With Prior Work

To the best of our knowledge, this is the first meta-analysis of RCTs that evaluates the effectiveness and safety of technology-based interventions for oral anticoagulation management. In published systematic meta-analyses and reviews, most of the literature evaluating the effects of technology-based interventions has focused on atrial fibrillation [34] and cancer [36]. Most studies regarding the administration of OACs have focused on the effects of managers on the effectiveness and safety of anticoagulation management, such as the differences between pharmacist management and physician management or between patient self-management and anticoagulation clinic management [37-41]. The intervention method we chose is a technology-based approach that might have also been included in other studies, such as pharmacist-managed or self-managed interventions [37,41].

Although the study of telemedicine is not specific to OACs and is primarily qualitative, previous studies have highlighted similar challenges [42-44]. Clinical pharmaceutical care provided by Niznik et al [43] through telemedicine (mainly by telephone) in inpatient or outpatient settings was found to have an overall positive impact on outcomes related to clinical disease management, patient self-management, and adherence to the management of various chronic diseases. The common ground in the studies that had a positive impact on outcomes was the use of continuous or regular patterns of care, including telephone interaction and frequent monitoring and intervention [43]. Xia et al’s [44] study was conducted to explore the effects of online and offline management of anticoagulants on therapeutic efficacy and adverse reactions. Considering the convenience and economy of technology-based interventions, online anticoagulation management is more suitable for patients with stable conditions and for whom transportation may be difficult, such as those with disabilities or who live far from the hospital. Lee et al’s study [42] suggested that most outcomes for telemedicine were similar to those for conventional medical care, but the incidence of major thromboembolism events was significantly lower in the telemedicine group. However, the papers they included had a higher risk of bias and were of a lower quality study design, and the level of evidence supporting this conclusion was very low.

### Conclusions

This meta-analysis explored the effects of technology-based interventions on oral anticoagulation management. The results demonstrate that the technology-based intervention group had significantly improved TTR compared with the traditional intervention group and that there were no significant differences between the 2 intervention models in terms of time within expanded target INR range and the incidence of major bleeding events, minor bleeding events, thromboembolic events, mortality, and hospitalization. Of the different management options, telephone intervention was found to be the most widely used and most convenient means of technology-based interventions; it enables patients to get a professional reply quickly and is not restricted by the patient’s ability to use the internet. OAC management through technology-based intervention appears to be superior to OAC management through traditional intervention and may provide more convenient and higher quality anticoagulation services for patients. Further research is needed to explore more optimal technology-based interventions in oral anticoagulation management in a wider array of health care settings.

## Appendix

Multimedia Appendix 1 Search strategy to find literature on the use of technology-based interventions for oral anticoagulation management.

## Abbreviations

eHealth: electronic health
INR: international normalized ratio
MD: mean difference
mHealth: mobile health
OAC: oral anticoagulant
RCT: randomized controlled trial
TTR: time in therapeutic range

## References

[1] Ruff Christian T, Giugliano Robert P, Braunwald Eugene, Hoffman Elaine B, Deenadayalu Naveen, Ezekowitz Michael D, Camm A John, Weitz Jeffrey I, Lewis Basil S, Parkhomenko Alexander, Yamashita Takeshi, Antman Elliott M (2014). Comparison of the efficacy and safety of new oral anticoagulants with warfarin in patients with atrial fibrillation: a meta-analysis of randomised trials. Lancet.

[2] Chatterjee Saurav, Sardar Partha, Biondi-Zoccai Giuseppe, Kumbhani Dharam J (2013). New oral anticoagulants and the risk of intracranial hemorrhage: traditional and Bayesian meta-analysis and mixed treatment comparison of randomized trials of new oral anticoagulants in atrial fibrillation. JAMA Neurol.

[3] Wardrop Douglas, Keeling David (2008). The story of the discovery of heparin and warfarin. Br J Haematol.

[4] Lee Ming Ta Michael, Klein Teri E (2013). Pharmacogenetics of warfarin: challenges and opportunities. J Hum Genet.

[5] Shah Zubair, Masoomi Reza, Tadros Peter (2015). Managing Antiplatelet Therapy and Anticoagulants in Patients with Coronary Artery Disease and Atrial Fibrillation. J Atr Fibrillation.

[6] Spyropoulos Alex C, Douketis James D (2012). How I treat anticoagulated patients undergoing an elective procedure or surgery. Blood.

[7] Douketis James D, Spyropoulos Alex C, Spencer Frederick A, Mayr Michael, Jaffer Amir K, Eckman Mark H, Dunn Andrew S, Kunz Regina (2012). Perioperative management of antithrombotic therapy: Antithrombotic Therapy and Prevention of Thrombosis, 9th ed: American College of Chest Physicians Evidence-Based Clinical Practice Guidelines. Chest.

[8] Viswanathan Meera, Golin Carol E, Jones Christine D, Ashok Mahima, Blalock Susan J, Wines Roberta C M, Coker-Schwimmer Emmanuel J L, Rosen David L, Sista Priyanka, Lohr Kathleen N (2012). Interventions to improve adherence to self-administered medications for chronic diseases in the United States: a systematic review. Ann Intern Med.

[9] Chaet D, Clearfield R, Sabin JE, Skimming K, Council on Ethical and Judicial Affairs American Medical Association (2017). Ethical practice in Telehealth and Telemedicine. J Gen Intern Med.

[10] Nelson Roxanne (2017). Telemedicine and Telehealth: The Potential to Improve Rural Access to Care. Am J Nurs.

[11] Krumm H, Reiss N, Burkert M, Schmidt T, Biehs S, Bohr C, Gurtler F, Horn H, Kreutzer P, Mewes P, Miller H, Riest C, Romer C, Seebold A, Sprung G, Ziegler O (2018). Development of a Computer-Aided Dosage and Telemonitoring System for Patients Under Oral Anticoagulation Therapy. Stud Health Technol Inform.

[12] Spyropoulos Alex C, Myrka Anne, Triller Darren M, Ragan Stephen, York Collin, King Jaz-Michael, Lee Ti-Kuang (2018). Uptake and Utilization of the Management of Anticoagulation in the Periprocedural Period App: Longitudinal Analysis. JMIR Mhealth Uhealth.

[13] Brasen Claus L, Madsen Jonna S, Parkner Tina, Brandslund Ivan (2019). Home Management of Warfarin Treatment Through a Real-Time Supervised Telemedicine Solution: A Randomized Controlled Trial. Telemed J E Health.

[14] Kane J M (2014). Technology-based interventions in health care. Epidemiol Psychiatr Sci.

[15] Deeken Friederike, Rezo Anna, Hinz Matthias, Discher Robert, Rapp Michael A (2019). Evaluation of Technology-Based Interventions for Informal Caregivers of Patients With Dementia-A Meta-Analysis of Randomized Controlled Trials. Am J Geriatr Psychiatry.

[16] Seaburg Luke, Hess Erik P, Coylewright Megan, Ting Henry H, McLeod Christopher J, Montori Victor M (2014). Shared decision making in atrial fibrillation: where we are and where we should be going. Circulation.

[17] Higgins Julian P T, Altman Douglas G, GØtzsche Peter C, Jüni Peter, Moher David, Oxman Andrew D, Savovic Jelena, Schulz Kenneth F, Weeks Laura, Sterne Jonathan A C, Cochrane Bias Methods Group, Cochrane Statistical Methods Group (2011). The Cochrane Collaboration's tool for assessing risk of bias in randomised trials. BMJ.

[18] Jin Yin Z, Yan Shi, Yuan Wen X (2017). Effect of isometric handgrip training on resting blood pressure in adults: a meta-analysis of randomized controlled trials. J Sports Med Phys Fitness.

[19] Beyth R J, Quinn L, Landefeld C S (2000). A multicomponent intervention to prevent major bleeding complications in older patients receiving warfarin. A randomized, controlled trial. Ann Intern Med.

[20] Sidhu P, O'Kane H O (2001). Self-managed anticoagulation: results from a two-year prospective randomized trial with heart valve patients. Ann Thorac Surg.

[21] Fitzmaurice D A, Murray E T, Gee K M, Allan T F, Hobbs F D R (2002). A randomised controlled trial of patient self management of oral anticoagulation treatment compared with primary care management. J Clin Pathol.

[22] Khan Tayyaba Irfan, Kamali Farhad, Kesteven Patrick, Avery Peter, Wynne Hilary (2004). The value of education and self-monitoring in the management of warfarin therapy in older patients with unstable control of anticoagulation. Br J Haematol.

[23] Staresinic Anthony G, Sorkness Christine A, Goodman Brian M, Pigarelli Denise Walbrandt (2006). Comparison of outcomes using 2 delivery models of anticoagulation care. Arch Intern Med.

[24] Chan Fredric W H, Wong Raymond S M, Lau Wing-Hung, Chan Thomas Y K, Cheng Gregory, You Joyce H S (2006). Management of Chinese patients on warfarin therapy in two models of anticoagulation service - a prospective randomized trial. Br J Clin Pharmacol.

[25] Lalonde Lyne, Martineau Josée, Blais Normand, Montigny Martine, Ginsberg Jeffrey, Fournier Martine, Berbiche Djamal, Vanier Marie-Claude, Blais Lucie, Perreault Sylvie, Rodrigues Isabel (2008). Is long-term pharmacist-managed anticoagulation service efficient? A pragmatic randomized controlled trial. Am Heart J.

[26] Soliman Hamad Mohamed A, van Eekelen Ellen, van Agt Ton, van Straten Albert H M (2009). Self-management program improves anticoagulation control and quality of life: a prospective randomized study. Eur J Cardiothorac Surg.

[27] Schillig Jessica, Kaatz Scott, Hudson Michael, Krol Gregory D, Szandzik Edward G, Kalus James S (2011). Clinical and safety impact of an inpatient pharmacist-directed anticoagulation service. J Hosp Med.

[28] Verret Lucie, Couturier Justine, Rozon Andréanne, Saudrais-Janecek Sarah, St-Onge Amélie, Nguyen Angela, Basmadjian Arsène, Tremblay Simon, Brouillette Denis, de Denus Simon (2012). Impact of a pharmacist-led warfarin self-management program on quality of life and anticoagulation control: a randomized trial. Pharmacotherapy.

[29] Bungard Tammy J, Ritchie Bruce, Garg Sipi, Tsuyuki Ross T (2012). Sustained impact of anticoagulant control achieved in an anticoagulation management service after transfer of management to the primary care physician. Pharmacotherapy.

[30] Lakshmi R, James E, Kirthivasan R (2013). Study on Impact of Clinical Pharmacist's Interventions in the Optimal Use of Oral Anticoagulants in Stroke Patients. Indian J Pharm Sci.

[31] Sudas Na Ayutthaya Natthaporn, Sakunrak Itsarawan, Dhippayom Teerapon (2018). Clinical Outcomes of Telemonitoring for Patients on Warfarin after Discharge from Hospital. Int J Telemed Appl.

[32] Liang Jia-Bi, Lao Cheng-Kin, Tian Lin, Yang Ying-Ying, Wu Hui-Min, Tong Henry Hoi-Yee, Chan Alexandre (2020). Impact of a pharmacist-led education and follow-up service on anticoagulation control and safety outcomes at a tertiary hospital in China: a randomised controlled trial. Int J Pharm Pract.

[33] Tideman Philip A, Tirimacco Rosy, St John Andrew, Roberts Gregory W (2015). How to manage warfarin therapy. Aust Prescr.

[34] Giebel Godwin Denk, Gissel Christian (2019). Accuracy of mHealth Devices for Atrial Fibrillation Screening: Systematic Review. JMIR Mhealth Uhealth.

[35] Ferrando Fernando, Mira Yolanda (2015). Effective and Safe Management of Oral Anticoagulation Therapy in Patients Who Use the Internet-Accessed Telecontrol Tool SintromacWeb. Interact J Med Res.

[36] Agboola Stephen O, Ju Woong, Elfiky Aymen, Kvedar Joseph C, Jethwani Kamal (2015). The effect of technology-based interventions on pain, depression, and quality of life in patients with cancer: a systematic review of randomized controlled trials. J Med Internet Res.

[37] Hou Kelu, Yang Hui, Ye Zhikang, Wang Ying, Liu Lihong, Cui Xiangli (2017). Effectiveness of Pharmacist-led Anticoagulation Management on Clinical Outcomes: A Systematic Review and Meta-Analysis. J Pharm Pharm Sci.

[38] Manzoor Beenish S, Cheng Wei-Han, Lee James C, Uppuluri Ellen M, Nutescu Edith A (2017). Quality of Pharmacist-Managed Anticoagulation Therapy in Long-Term Ambulatory Settings: A Systematic Review. Ann Pharmacother.

[39] Wofford James L, Wells Megan D, Singh Sonal (2008). Best strategies for patient education about anticoagulation with warfarin: a systematic review. BMC Health Serv Res.

[40] Xu Zhe, Wang Zhiping, Ou Jingsong, Xu Yingqi, Yang Song, Zhang Xi (2012). Two monitoring methods of oral anticoagulant therapy in patients with mechanical heart valve prothesis: a meta-analysis. J Thromb Thrombolysis.

[41] Zhou S, Sheng X Y, Xiang Q, Wang Z N, Zhou Y, Cui Y M (2016). Comparing the effectiveness of pharmacist-managed warfarin anticoagulation with other models: a systematic review and meta-analysis. J Clin Pharm Ther.

[42] Lee Munil, Wang Mei, Liu Jiayu, Holbrook Anne (2018). Do telehealth interventions improve oral anticoagulation management? A systematic review and meta-analysis. J Thromb Thrombolysis.

[43] Niznik Joshua D, He Harvey, Kane-Gill Sandra L (2018). Impact of clinical pharmacist services delivered via telemedicine in the outpatient or ambulatory care setting: A systematic review. Res Social Adm Pharm.

[44] Xia Xiaotong, Wu Jianmei, Zhang Jinhua (2018). The effect of online versus hospital warfarin management on patient outcomes: a systematic review and meta-analysis. Int J Clin Pharm.

