# Correction: Technology-Based Interventions in Oral Anticoagulation Management: Meta-Analysis of Randomized Controlled Trials

**DOI:** 10.2196/22761

**Published:** 2020-08-04

**Authors:** Hengfen Dai, Caiyun Zheng, Chun Lin, Yan Zhang, Hong Zhang, Fan Chen, Yunchun Liu, Jingwen Xiao, Chaoxin Chen

**Affiliations:** 1 Affiliated Fuzhou First Hospital of Fujian Medical University Fuzhou China; 2 Fuqing City Hospital Fuzhou China; 3 School of Basic Medical Sciences Fujian Medical University Fuzhou China; 4 School of Pharmacy Fujian Medical University Fuzhou China

In “Technology-Based Interventions in Oral Anticoagulation Management: Meta-Analysis of Randomized Controlled Trials” (JMIR 2020;22(7):e18386) the authors noted an error in the order of authorship. The authors wish to change the order of the first and second authors so that Hengfen Dai is first author and Caiyun Zheng is second author.

The previous order of authorship was as follows:

Caiyun Zheng, Hengfen Dai, Chun Lin, Yan Zhang, Hong Zhang, Fan Chen, Yunchun Liu, Jingwen Xiao, Chaoxin Chen

The correct order of authorship is as follows:

Hengfen Dai, Caiyun Zheng, Chun Lin, Yan Zhang, Hong Zhang, Fan Chen, Yunchun Liu, Jingwen Xiao, Chaoxin Chen

Additionally, affiliations have been reordered in accordance with the correct authorship order. Affiliations were previously listed as:

^1^Fuqing City Hospital, Fuzhou, China

^2^Affiliated Fuzhou First Hospital of Fujian Medical University, Fuzhou, China

^3^School of Basic Medical Sciences, Fujian Medical University, Fuzhou, China

^4^School of Pharmacy, Fujian Medical University, Fuzhou, China

The revised order of affiliations is:

^1^Affiliated Fuzhou First Hospital of Fujian Medical University, Fuzhou, China

^2^Fuqing City Hospital, Fuzhou, China

^3^School of Basic Medical Sciences, Fujian Medical University, Fuzhou, China

^4^School of Pharmacy, Fujian Medical University, Fuzhou, China

Hengfen Dai, Caiyun Zheng, and Chun Lin remain credited with equal contribution.

The correction will appear in the online version of the paper on the JMIR Publications website on August 4, 2020, together with the publication of this correction notice. Because this was made after submission to PubMed, PubMed Central, and other full-text repositories, the corrected article has also been resubmitted to those repositories.

