# Supplementary material for: Technology-Based Interventions in Oral Anticoagulation Management: Meta-Analysis of Randomized Controlled Trials
Source: J Med Internet Res. 2020 Jul 15;22(7):e18386. doi: 10.2196/18386 (PMC7391164; doi:10.2196/18386)
Supplement: Multimedia Appendix 1 [file jmir_v22i7e18386_app1.doc]

Appendix I

**1361Pubmed:**

#1: (anticoagulants[mesh terms]) or (Anticoagulation Agents) or (Agents, Anticoagulation) or (Anticoagulant Agents) or (Agents, Anticoagulant) or (Anticoagulant Drugs) or (Drugs, Anticoagulant) or (Anticoagulant) or (Indirect Thrombin Inhibitors) or (Inhibitors, Indirect Thrombin) or (Thrombin Inhibitors, Indirect) or (anticoagulant) or (decoagulant) or (antithrombotics) or (anticaking agent) or (anticoagulation) or (anticoagulation therapy) or (anticoagulation treatment) or (anticoagulation management) or (anticoagulation services)

#2: (warfarin[mesh terms]) or (4-Hydroxy-3-(3-oxo-1-phenylbutyl)-2H-1-benzopyran-2-one) or

(Apo-Warfarin) or (Aldocumar) or (Gen-Warfarin) or (Warfant) or (Coumadin) or (Marevan) or (Warfarin Potassium) or (Potassium, Warfarin) or (Warfarin Sodium) or (Sodium, Warfarin) or (Coumadine) or (Tedicumar) or (warfarin therapy)

#3: (Telemedicine[mesh terms]) or (Mobile Health) or (Health, Mobile) or (mHealth) or (Telehealth) or (eHealth) or (connected health) or (text messaging) or (telephone therapy) or (teleconsultation) or (mobile technology) or (telecare) or (Internet) or (digital health) or (mobile phone) or (smartphone) or (apps) or ("Mobile Applications"[Mesh terms]) OR (Application, Mobile) OR (Applications, Mobile) OR (Mobile Application) OR (Mobile Apps App, Mobile) OR (Apps, Mobile) OR (Mobile App) OR (Portable Electronic Apps) OR (App, Portable Electronic) OR (Apps, Portable Electronic) OR (Electronic App, Portable) OR (Electronic Apps, Portable) OR (Portable Electronic App) OR (Portable Electronic Applications) OR (Application, Portable Electronic) OR (Applications, Portable Electronic) OR (Electronic Application, Portable) OR (Electronic Applications, Portable) OR (Portable Electronic Application) OR (Portable Software Apps) OR (App, Portable Software) OR (Apps, Portable Software) OR (Portable Software App) OR (Software App, Portable) OR (Software Apps, Portable) OR (Portable Software Applications) OR (Application, Portable Software) OR (Applications, Portable Software) OR (Portable Software Application) OR (Software Application, Portable) OR (Software Applications, Portable)

#4:#1 or #2

#5:#4 and #3

**2775Embase:**

#1: 'anticoagulation'/exp OR (anticoagulation AND agents) OR (agents, AND anticoagulation) OR (anticoagulant AND agents) OR (agents, AND anticoagulant) OR (anticoagulant AND drugs) OR (drugs, AND anticoagulant) OR (indirect AND thrombin AND inhibitors) OR (inhibitors, AND indirect AND thrombin) OR (thrombin AND inhibitors, AND indirect) OR anticoagulant OR decoagulant OR antithrombotics OR (anticaking AND agent) OR anticoagulation OR (anticoagulation AND therapy) OR (anticoagulation AND treatment) OR (anticoagulation AND management) OR (anticoagulation AND services)

#2: 'warfarin'/exp OR '4 hydroxy 3 3 oxo 1 phenylbutyl 2h 1 benzopyran 2 one' OR 'apo warfarin' OR aldocumar OR 'gen warfarin' OR warfant OR coumadin OR marevan OR (warfarin AND potassium) OR (potassium, AND warfarin) OR (warfarin AND sodium) OR (sodium, AND warfarin) OR coumadine OR tedicumar OR (warfarin AND therapy)

#3:#1 or #2

#4: 'telemedicine'/exp OR (mobile AND health) OR (health, AND mobile) OR mhealth OR telehealth OR ehealth OR (connected AND health) OR (text AND messaging) OR (telephone AND therapy) OR teleconsultation OR (mobile AND technology) OR telecare OR internet OR (digital AND health) OR (mobile AND phone) OR smartphone OR apps

#5: 'mobile application'/exp OR (application, AND mobile) OR (applications, AND mobile) OR (mobile AND application) OR (apps AND app, AND mobile) OR (apps, AND mobile) OR (mobile AND app) OR (portable AND electronic AND apps) OR (app, AND portable AND electronic) OR (apps, AND portable AND electronic) OR (electronic AND app, AND portable) OR (electronic AND apps, AND portable) OR (portable AND electronic AND app) OR (portable AND electronic AND applications) OR (application, AND portable AND electronic) OR (applications, AND portable AND electronic) OR (electronic AND application, AND portable) OR (electronic AND applications, AND portable) OR (portable AND electronic AND application) OR (portable AND software AND apps) OR (app, AND portable AND software) OR (apps, AND portable AND software) OR (portable AND software AND app) OR (software AND app, AND portable) OR (software AND apps, AND portable) OR (portable AND software AND applications) OR (application, AND portable AND software) OR (applications, AND portable AND software) OR (portable AND software AND application) OR (software AND application, AND portable) OR (software AND applications, AND portable)

#6:#4 or #5

#7:#3 and #6

159[**the Cochrane Library**](http://www.baidu.com/link?url=ajG4RuqShux6nlg5oTRROhYeepvQXFzvRL5tshPuX6zmvs4bhDWv9Hs5W1lTPi3B8inUJlqu2pk7fz2CClGFRESr255lRCshyLvCoiYaF2S)

**#1:** (anticoagulants[mh]) or (Anticoagulation Agents) or (Agents, Anticoagulation) or (Anticoagulant Agents) or (Agents, Anticoagulant) or (Anticoagulant Drugs) or (Drugs, Anticoagulant) or (Anticoagulant) or (Indirect Thrombin Inhibitors) or (Inhibitors, Indirect Thrombin) or (Thrombin Inhibitors, Indirect) or (anticoagulant) or (decoagulant) or (antithrombotics) or (anticaking agent) or (anticoagulation) or (anticoagulation therapy) or (anticoagulation treatment) or (anticoagulation management) or (anticoagulation services)

#2: (warfarin[mh]) or (Apo-Warfarin) or (Aldocumar) or (Gen-Warfarin) or (Warfant) or (Coumadin) or (Marevan) or (Warfarin Potassium) or (Potassium, Warfarin) or (Warfarin Sodium) or (Sodium, Warfarin) or (Coumadine) or (Tedicumar) or (warfarin therapy)

**#3:** (Telemedicine[mh]) or (Mobile Health) or (Health, Mobile) or (mHealth) or (Telehealth) or (eHealth) or (connected health) or (text messaging) or (telephone therapy) or (teleconsultation) or (mobile technology) or (telecare) or (Internet) or (digital health) or (mobile phone) or (smartphone) or (apps) or ("Mobile Applications"[mh]) OR (Application, Mobile) OR (Applications, Mobile) OR (Mobile Application) OR (Mobile Apps App, Mobile) OR (Apps, Mobile) OR (Mobile App) OR (Portable Electronic Apps) OR (App, Portable Electronic) OR (Apps, Portable Electronic) OR (Electronic App, Portable) OR (Electronic Apps, Portable) OR (Portable Electronic App) OR (Portable Electronic Applications) OR (Application, Portable Electronic) OR (Applications, Portable Electronic) OR (Electronic Application, Portable) OR (Electronic Applications, Portable) OR (Portable Electronic Application) OR (Portable Software Apps) OR (App, Portable Software) OR (Apps, Portable Software) OR (Portable Software App) OR (Software App, Portable) OR (Software Apps, Portable) OR (Portable Software Applications) OR (Application, Portable Software) OR (Applications, Portable Software) OR (Portable Software Application) OR (Software Application, Portable) OR (Software Applications, Portable)

**84CINAHL**

**#1:** (MM " warfarin") or (Apo-Warfarin) or (Aldocumar) or (Gen-Warfarin) or (Warfant) or (Coumadin) or (Marevan) or (Warfarin Potassium) or (Potassium, Warfarin) or (Warfarin Sodium) or (Sodium, Warfarin) or (Coumadine) or (Tedicumar) or (warfarin therapy) or (MM " anticoagulants") or (Anticoagulation Agents) or (Agents, Anticoagulation) or (Anticoagulant Agents) or (Agents, Anticoagulant) or (Anticoagulant Drugs) or (Drugs, Anticoagulant) or (Anticoagulant) or (Indirect Thrombin Inhibitors) or (Inhibitors, Indirect Thrombin) or (Thrombin Inhibitors, Indirect) or (anticoagulant) or (decoagulant) or (antithrombotics) or (anticaking agent) or (anticoagulation) or (anticoagulation therapy) or (anticoagulation treatment) or (anticoagulation management) or (anticoagulation services)

#2: (MM"Telemedicine") or (Mobile Health) or (Health, Mobile) or (mHealth) or (Telehealth) or (eHealth) or (connected health) or (text messaging) or (telephone therapy) or (teleconsultation) or (mobile technology) or (telecare) or (Internet) or (digital health) or (mobile phone) or (smartphone) or (apps) or (MM"Mobile Applications") OR (Application, Mobile) OR (Applications, Mobile) OR (Mobile Application) OR (Mobile Apps App, Mobile) OR (Apps, Mobile) OR (Mobile App) OR (Portable Electronic Apps) OR (App, Portable Electronic) OR (Apps, Portable Electronic) OR (Electronic App, Portable) OR (Electronic Apps, Portable) OR (Portable Electronic App) OR (Portable Electronic Applications) OR (Application, Portable Electronic) OR (Applications, Portable Electronic) OR (Electronic Application, Portable) OR (Electronic Applications, Portable) OR (Portable Electronic Application) OR (Portable Software Apps) OR (App, Portable Software) OR (Apps, Portable Software) OR (Portable Software App) OR (Software App, Portable) OR (Software Apps, Portable) OR (Portable Software Applications) OR (Application, Portable Software) OR (Applications, Portable Software) OR (Portable Software Application) OR (Software Application, Portable) OR (Software Applications, Portable)

#3:#1 and #2

**405PsycINFO**

(("MM warfarin" or Apo-Warfarin or Aldocumar or Gen-Warfarin or Warfant or Coumadin or Marevan or Warfarin Potassium or Potassium, Warfarin or Warfarin Sodium or Sodium, Warfarin or Coumadine or Tedicumar or warfarin therapy or "MM anticoagulants" or Anticoagulation Agents or Agents, Anticoagulation or Anticoagulant Agents or Agents, Anticoagulant or Anticoagulant Drugs or Drugs, Anticoagulant or Anticoagulant or Indirect Thrombin Inhibitors or Inhibitors, Indirect Thrombin or Thrombin Inhibitors, Indirect or anticoagulant or decoagulant or antithrombotics or anticaking agent or anticoagulation or anticoagulation therapy or anticoagulation treatment or anticoagulation management or anticoagulation services) and ("MM Telemedicine" or Mobile Health or Health, Mobile or mHealth or Telehealth or eHealth or connected health or text messaging or telephone therapy or teleconsultation or mobile technology or telecare or Internet or digital health or mobile phone or smartphone or apps or "MM Mobile Applications" or Application, Mobile or Applications, Mobile or Mobile Application or Mobile Apps App, Mobile or Apps, Mobile or Mobile App or Portable Electronic Apps or App, Portable Electronic or Apps, Portable Electronic or Electronic App, Portable or Electronic Apps, Portable or Portable Electronic App or Portable Electronic Applications or Application, Portable Electronic or Applications, Portable Electronic or Electronic Application, Portable or Electronic Applications, Portable or Portable Electronic Application or Portable Software Apps or App, Portable Software or Apps, Portable Software or Portable Software App or Software App, Portable or Software Apps, Portable or Portable Software Applications or Application, Portable Software or Applications, Portable Software or Portable Software Application or Software Application, Portable or Software Applications, Portable)).af.

**2000 Scopus-6752**

(( anticoagulants[mesh terms]) or (anticoagulation) or (anticoagulation therapy) or (anticoagulation treatment) or (anticoagulation management) or (anticoagulation services) or (warfarin[mesh terms]) or (warfarin therapy)) AND ((Telemedicine[mesh terms]) or (Mobile Health) or (Health, Mobile) or (mHealth) or (Telehealth) or (eHealth) or (connected health) or (text messaging) or (telephone therapy) or (teleconsultation) or (mobile technology) or (telecare) or (Internet) or (digital health) or (mobile phone) or (smartphone) or (apps) )
